# Evidence and Consequence of a Highly Adapted Clonal Haplotype within the Australian *Ascochyta rabiei* Population

**DOI:** 10.3389/fpls.2017.01029

**Published:** 2017-06-16

**Authors:** Yasir Mehmood, Prabhakaran Sambasivam, Sukhjiwan Kaur, Jenny Davidson, Audrey E. Leo, Kristy Hobson, Celeste C. Linde, Kevin Moore, Jeremy Brownlie, Rebecca Ford

**Affiliations:** ^1^Environmental Futures Research Institute, School of Natural Sciences, Griffith University, NathanQLD, Australia; ^2^Agriculture Victoria, AgriBio, The Centre for AgriBioscience, BundooraVIC, Australia; ^3^South Australian Research and Development Institute, UrrbraeSA, Australia; ^4^New South Wales Department of Primary Industries, Wagga Wagga Agricultural Institute, Wagga WaggaNSW, Australia; ^5^Department of Primary Industries, Tamworth Agricultural Institute, TamworthNSW, Australia; ^6^Research School of Biology, Australian National University, CanberraACT, Australia

**Keywords:** chickpea, *Ascochyta rabiei*, resistance sources, SSR genotype, haplotype and highly aggressive

## Abstract

The Australian *Ascochyta rabiei* (Pass.) Labr. (syn. *Phoma rabiei*) population has low genotypic diversity with only one mating type detected to date, potentially precluding substantial evolution through recombination. However, a large diversity in aggressiveness exists. In an effort to better understand the risk from selective adaptation to currently used resistance sources and chemical control strategies, the population was examined in detail. For this, a total of 598 isolates were quasi-hierarchically sampled between 2013 and 2015 across all major Australian chickpea growing regions and commonly grown host genotypes. Although a large number of haplotypes were identified (66) through short sequence repeat (SSR) genotyping, overall low gene diversity (*H_exp_* = 0.066) and genotypic diversity (*D* = 0.57) was detected. Almost 70% of the isolates assessed were of a single dominant haplotype (ARH01). Disease screening on a differential host set, including three commonly deployed resistance sources, revealed distinct aggressiveness among the isolates, with 17% of all isolates identified as highly aggressive. Almost 75% of these were of the ARH01 haplotype. A similar pattern was observed at the host level, with 46% of all isolates collected from the commonly grown host genotype Genesis090 (classified as “resistant” during the term of collection) identified as highly aggressive. Of these, 63% belonged to the ARH01 haplotype. In conclusion, the ARH01 haplotype represents a significant risk to the Australian chickpea industry, being not only widely adapted to the diverse agro-geographical environments of the Australian chickpea growing regions, but also containing a disproportionately large number of aggressive isolates, indicating fitness to survive and replicate on the best resistance sources in the Australian germplasm.

## Introduction

Chickpea (*Cicer arietinum* L.), is the most widely cultivated legume, grown in over 50 countries across the Indian subcontinent, North Africa, the Middle East, southern Europe, the Americas and Australia. The global production in 2014 was 14 million tons with yields of^1^ 982 kg/ha. The crop is grown in rotation, largely for its high cash return and ability to fix atmospheric nitrogen (Gan et al., 2006). However, significant yield instability remains, largely due to Ascochyta blight caused by the necrotrophic fungal pathogen *Ascochyta rabiei* (Nene, 1982). The disease causes extensive crop losses globally (Pande et al., 2005), and remains the major biotic constraint to the winter-grown crop in Australia, with all growing regions affected (Bretag et al., 2008). Subsequent inoculum release following increased precipitation over the 2013 to 2016 growing seasons has led to non-manageable epidemics on “resistant” host genotypes (Moore et al., 2016). The recent severity of the disease is likely due to the dispersal of isolates that are highly aggressive, widely adapted and able to survive between the growing seasons in the harsh Australian summer climate.

*Ascochyta rabiei* is a bipolar heterothallic fungus with one mating type locus and two mating types (Wilson and Kaiser, 1995). Large temporal and spatial variations have been detected within populations from other global regions where both mating types exist (Udupa et al., 1998; Jamil et al., 2000; Peever et al., 2004; Ali et al., 2012). On a global scale, the total gene diversity detected with 19 sequence tag microsatellite primers was estimated to be 0.29 among *A. rabiei* populations worldwide. Maximum gene diversity was detected among intra-country populations in Canada (0.38), followed by the United States (0.36) and Syria (0.32) (Phan et al., 2003). In other studies, based on different sets of short sequence repeat (SSR) loci, the diversity of the population was estimated to be even higher; 0.55 in Tunisia (Rhaïem et al., 2006), and 0.79 in Iran (Nourollahi et al., 2011). This is in stark contrast to the population diversity observed in Australia, where despite trapping of the putative ascospore in the field (Galloway and MacLeod, 2003), only one mating type has been detected (Barve et al., 2003; Leo et al., 2015). Accordingly, multiple studies have shown a very low gene diversity within the population (ranging from 0.02 to 0.094), consistent with an organism that is reproducing asexually (Phan et al., 2003; Rhaïem et al., 2006; Leo et al., 2015).

The variation in aggressiveness detected within sexually recombinant *A. rabiei* populations worldwide has led to the erosion of resistant host genotypes (McDonald and Linde, 2002; Peever et al., 2012; Mahiout et al., 2015; Vafaei et al., 2015; Tekin et al., 2017). Although not directly comparable due to a number of factorial differences such as host genotype, isolate and bioassay conditions, several in country studies have identified diversity of aggressiveness within *A. rabiei* populations. Sets of isolates have been identified that react similarly or differently to a group of host genotypes (Grewal, 1984; Udupa et al., 1998; Jayakumar et al., 2005; Pande et al., 2005; Imtiaz et al., 2015; Vafaei et al., 2015; Baite et al., 2016). Jan and Wiese (1991) reported the presence of 11 “virulent forms” among 39 isolates assessed from the Palouse region of the United States. Navas-Cortés et al. (1998) identified 11 “pathotypes” in India, Pakistan, Spain, and the United States. Next, Jamil et al. (2000) classified 102 isolates from Pakistan into eight virulence forms and 14 “pathotype groups” were identified among 40 Canadian isolates assessed for disease reaction on eight chickpea differential lines (Chongo et al., 2004). Pouralibaba et al. (2008) reported three “pathotype groups” present in north-western Iran, whereas Ghiai et al. (2012) reported 10 “virulent forms” and 16 “pathogenic groups,” respectively, from Iran. Most recently, a new highly virulent “pathotype IV” was reported in Syria and the existence of the four previously identified Syrian pathotypes (Atik et al., 2013) were confirmed (Imtiaz et al., 2015).

In Australia, although it appears that the population is largely clonal based on neutral genetic markers, a broad range of aggressiveness exists (Elliott et al., 2013). Hence the Australian chickpea industry is at risk from selective propagation and dispersal of the fittest and best adapted *A. rabiei* clones. Indeed, since host resistance is multigenic and partial (Cho et al., 2004), there is a heightened risk of resistance erosion caused by selection and increasing frequency of individual clones, with the ability to overcome singular or multiple defense genes/strategies as well as maintain peak fitness (Andrivon et al., 2007).

Two types of adaptation are recognized in fungal species, generalized adaptation and localized adaptation, both resulting in the production of unique haplotypes with high aggressiveness levels and frequencies, due directly to high survival rates (Leonard, 1977). Elliott et al. (2013) first detected clones with differing levels of aggressiveness within the Australian *A. rabiei* population and proposed that despite its clonal nature, the population contained a large potential to evolve and adapt to overcome chemical and host resistance management strategies. This proposal was based on a small number of isolates. To better understand and manage this risk a much larger study, encompassing a greater number of isolates from multiple growing regions and host genotypes collected over several growing seasons was required. This rationale is supported by observations over recent seasons of severe disease symptomology on host genotypes widely adopted throughout the Australian growing regions and that, until very recently, were considered “resistant” (in the case of Genesis 090 in the southern Victoria and South Australia regions) or “moderately resistant” (in the case of PBA HatTrick in the northern New South Wales and southern Queensland regions).

In order to assess risk to currently employed host resistance and chemical control strategies, as well as to better select for resistance longevity, an in depth understanding of the genetic and pathogenic structure of the Australian *A. rabiei* population is required. Therefore the aims of this study were to (1) assess the genetic structure of the *A. rabiei* population and any changes in the structure within and between the major chickpea growing regions of Australia and host genotypes sown, and (2) assess the spread and frequency of the most frequently occurring haplotypes containing the most aggressive isolates, to identify those isolates of highest risk to the Australian chickpea industry. Used together, this new knowledge of diversity, haplotype frequency and aggressiveness will enable strategic choice of isolates for application to resistance breeding programs and to assess for sustainability of resistance in newly deployed and soon to be widely adopted host genotypes.

## Materials and Methods

### Population Structure: SSRs

#### Isolate Collection and Culturing

**FIGURE 1:**
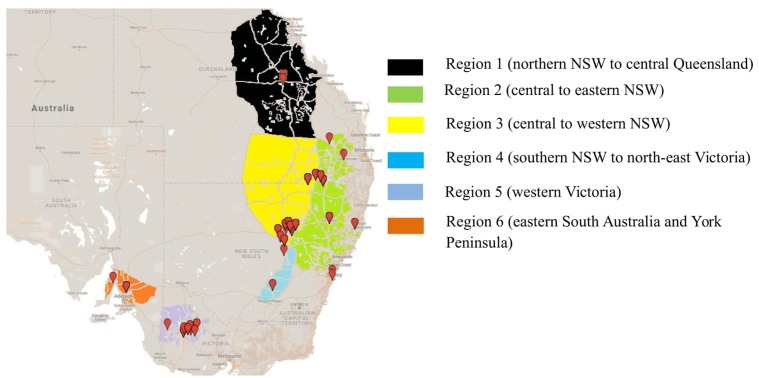
Isolate collection of *Ascochyta rabiei* from six different agro-geographically classified chickpea growing regions during three consecutive growing seasons (2013–2015).

To determine the structure of the Australian *A. rabiei* population, isolates were collected from commercial chickpea crops and National Variety Trial (NVT) sites during 2013–2015. This was done in a quasi-hierarchical manner, in that wherever possible, infected material was collected from the four corners and one central location within each field. At NVT sites, infected material was collected from as many host genotypes as possible, one sample from each genotype row at each location. For the overall Australian *A. rabiei* population study, a total of 598 isolates were collected from across the six agro-geographical chickpea growing regions in eastern and southern Australia (**Figure 1**). The full list of isolates and their available passport data (place of collection, year of collection, and host genotype) is provided in the additional material (Online source 1).

To assess for selective adaptation on widely grown Australian host genotypes, isolates were intensively collected from Genesis 090 and PBA HatTrick. At the time of study, these were rated as “resistant” and “moderately resistant,” respectively (Pulse Australia, 2009).

**FIGURE 2:**
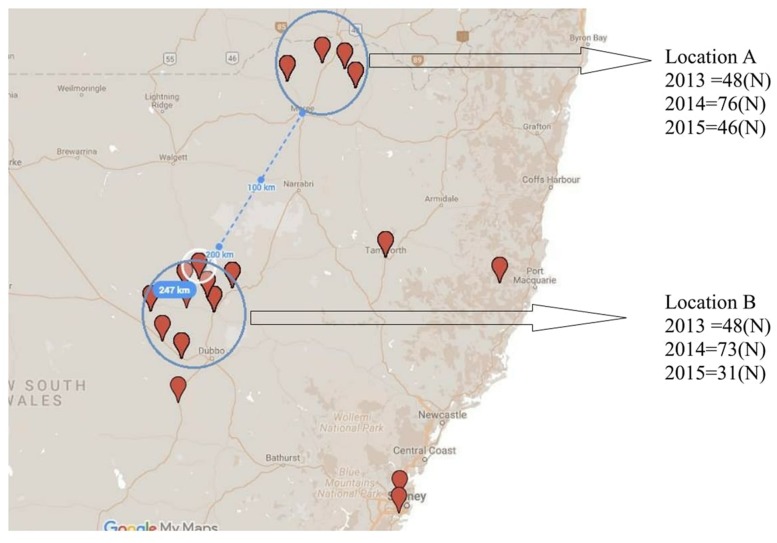
Collection locations and isolate numbers recovered (N) from PBA HatTrick over three consecutive years (2013–2015) for potential association of spatial and temporal effects.

In order to assess for effect of location and track any shift in population structure associated with a single host genotype over time, isolates were collected repeatedly over three consecutive growing seasons (2013, 2014, and 2015) from PBA HatTrick grown in the same two locations (locations A and B), each with a 50 km radius and 247 km apart (**Figure 2**).

Individual isolates were recovered from pycnidia of only one lesion per infected plant to minimize the likelihood of sampling clones due to short distance dispersal of conidia through rain splash. A single pycnidium per lesion was picked with a sterile needle from an infected chickpea leaf, stem or pod and inoculated into 2 mL of sterile distilled water before streaking onto V8 juice growth agar. Leaf lesions with no visible pycnidia were surface sterilized and placed on V8 juice growth agar. All agar plates were incubated for 14 days at 22 ± 2°C with a 12/12 h near-UV light irradiation (350–400 nm)/dark photoperiod, and resulting cultures were single spored on V8 juice agar media (Elliott et al., 2013).

#### DNA Extraction and SSR Genotyping

Five hundred and ninety-eight single spored isolates were inoculated separately into 25 mL falcon tubes containing Czapek Dox broth (Difco, Australia) and incubated for 2 weeks at 22 ± 2°C in the dark. Mycelia were then harvested and genomic DNA was extracted using the DNeasy Plant Mini Kit (Qiagen, United States) according to the manufacturer’s instructions.

Seven previously characterized and informative SSR loci were used for determining the genetic structure of the population (Leo et al., 2011). Genotyping was performed using the Multiplex-Ready PCR technique (Hayden et al., 2008), products were separated on a 96 capillary ABI 3730 DNA electrophoresis analyser and allele sizes were analyzed using GeneMapper v4.0 software (Applied Bio-systems) at the Australian Genome Research Facility (AGRF). Allele data was incorporated in population analysis of sizes only relevant to the previously characterized loci repeat polymorphisms.

#### Molecular Data and Population Structure Analysis

Number of alleles (Na), number of effective alleles (Ne) and Nei’s unbiased gene diversity (*H_exp_*) (Nei, 1978) was used to calculate the genetic diversity was calculated in GenAlex 6.5 (Peakall and Smouse, 2012). The number of multilocus genotypes (MLG), the number of expected MLGs at the smallest sample size based on rarefaction (*eMLG*), the corrected genotypic diversity index (*D*), MLG and genotypic evenness (*E.5*) were calculated using the Poppr package (Kamvar et al., 2014) in R (R Core Team, 2013). Analysis of molecular variance (AMOVA) was performed to examine the variation within and among the above mentioned sub-populations and multilocus analysis was performed to group isolates into haplotypes (online source 1) using GenAlex 6.5. (Peakall and Smouse, 2012).

To visualize the relationships among MLGs in the six sub-populations, SSR data were used to construct a minimum spanning network based on Bruvo’s distance (Bruvo et al., 2004) using the R package Poppr on non-clone-corrected data. The network was visualized using the package igraph (Csardi and Nepusz, 2006). Subsequently, the frequencies of most common haplotype were evaluated separately.

### Pathogenic Population Structure

#### Plant Material

Four chickpea genotypes with known disease reactions were used as a differential host set to assess isolate aggressiveness (**Table 1**). ICC3996 is used widely as a resistance source in the Australian chickpea breeding program, and Genesis090 and PBA HatTrick are the most widely grown “resistant” host genotypes in southern and northern regions, respectively. Meanwhile, Kyabra remains a widely grown host genotype in the harsher regions of New South Wales and southern and central regions of Queensland due to high yield and quality but is considered “susceptible” and used as a disease check in NVT sites. Seedlings were grown in 15 cm diameter pots containing commercial grade potting mix. Two replicates were sown for each of the genotype × isolate combinations assessed, with five plants grown per pot/rep. All plants were grown and maintained in the glasshouse facility at 22 ± 5°C under 16 h/8 h day/night photoperiod at The University of Melbourne, Parkville campus, Victoria, Australia.

**Table 1:** Differential host genotypes and their known disease response ratings to *A. rabiei* in Australia.

Genotype	Resistance level	Reference
Genesis 090 (kabuli)	R	Pulse Australia, 2017
ICC3996 (desi)	R	Nasir et al., 2000
PBA HatTrick (desi)	MR	Pulse Breeding Australia, 2017
Kyabra (desi)	S	Moore et al., 2015

#### Fungal Materials, Inoculation and Disease Assessment

Two hundred and sixty single spored *A. rabiei* isolates were selected for phenotyping, representative of the years, regions and host genotype origins within the 2013–2015 collection. This included sub-sets of isolates from targeted regions and genotypes as mentioned in section “Population Structure: SSRs”. Single spored isolates were cultured in V8 juice agar and maintained in the incubator for 14 days at 22 ± 2°C with a 12/12 h near-UV light irradiation (350–400 nm)/dark photoperiod prior to being used in the inoculation bioassay.

Inoculum was prepared as described in Sambasivam et al. (2017) and the mini-dome technique of Chen et al. (2005) was used to initiate disease. The disease severity of each isolate on each of the host genotypes was assessed using the qualitative 1–9 scale of Singh et al. (1981) at 21 days after inoculation (dai) where; scores of 1 or 3 represented a low disease severity; 5 represented a moderate disease severity without significant stem infection, and 7 or 9 represented a high disease severity with stem lesions that would lead to major difficulties in transpiration, photosynthesis and/or breakage.

#### Highly Aggressive Isolates and Pathogenicity Grouping

Isolates identified as highly aggressive produced a cumulative leaf score of at least 7 on >80% and a stem score of at least 7 on >10% of all of the host plants assessed. Subsequently, this sub-set of isolates were placed into pathogenicity groups based on their ability to cause low, moderate or high disease severity independently on ICC3996, Genesis090 and PBA HatTrick (**Table 2**).

**Table 2:** Criteria used for pathogenicity grouping of the highly aggressive isolates.

Pathogenicity group	Description
1	High disease on PBA HatTrick and low disease on Genesis090 and ICC3996
2	High disease incidence on PBA HatTrick, moderate disease on Genesis090 and low disease on ICC3996
3	High disease on PBA HatTrick, moderate disease on Genesis090 and moderate disease on ICC3996
4	High disease on PBA HatTrick, high disease on Genesis090 and moderate disease on ICC3996

#### Highest Risk Isolates to the Australian Chickpea Industry

Isolates of highest risk were identified on the basis of genotype and phenotype data. Accordingly, these belonged to the most frequently detected haplotype and were the most aggressive on the best resistance sources used within the advanced breeding program. Highest risk isolates were also aggressive on the currently deployed “resistant” host genotypes (pathogenicity group 4).

## Results

### Population Structure: SSRs

Between two and eight alleles were identified for each of the SSR loci across the collection of 598 isolates. The maximum gene diversity (*H_exp_*) at each ranged from 0.020 to 0.183 with an average of 0.066 (*H_exp_*). Locus ArA03T (*H_exp_* = 0.183) was the most informative, followed by ArH05T (*H_exp_* = 0.132) (**Table 3**).

**Table 3:** The informative microsatellite loci used for genotyping the Australian *A. rabiei* population.

Locus	Allele size	Number of allele	Number of Allele in regions						Size of allele	Diversity (*H_exp_*) ±SE
			Region 1 (*N* = 18)	Region 2 (*N* = 233)	Region 3 (*N* = 130)	Region 4 (*N* = 92)	Region 5 (*N* = 56)	Region 6 (*N* = 69)		
ArA03T	409–439	8	5	3	4	2	3	4	409, 412, 415, 421, 424, 427, 430, 433, 439	0.183 ± 0.057
ArH05T	221–257	6	2	4	2	3	2	3	221, 233, 239, 242, 248, 254	0.132 ± 0.035
ArR12D	185–191	3	2	1	2	2	2	2	185, 187, 189, 191	0.038 ± 0.017
ME14-1-56	379–383	2	2	1	1	1	1	1	379, 383	0.039 ± 0.039
ME14-1-63	313–319	3	1	2	1	3	2	1	313, 316, 319	0.033 ± 0.021
ME14-1-83	283–285	2	2	1	1	1	1	1	283, 285	0.020 ± 0.019
ME14-1-91	339-342	2	2	1	2	1	1	1	333, 339, 342	0.021 ± 0.018
						Mean (*H_exp_*) = 0.066 ± 0.015	

*N = Number of individuals observed. *H_exp_* = Nei’s unbiased gene diversity (Nei, 1978). SE = Standard error*.

**FIGURE 3:**
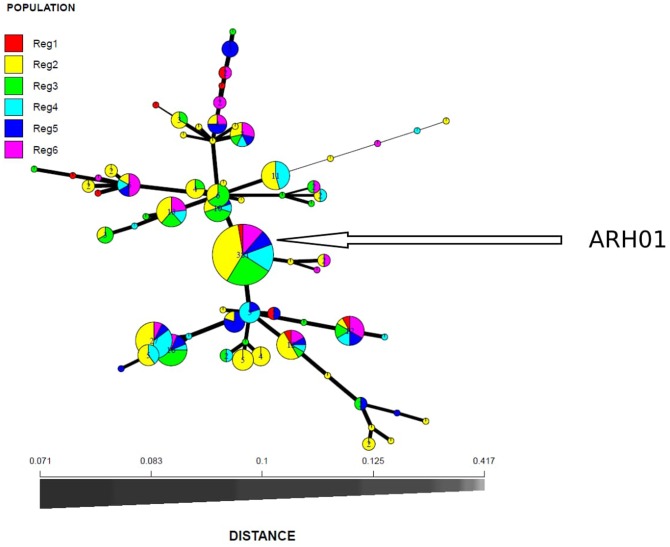
Minimum spanning network based on Bruvo genetic distances representing 66 MLGs observed in six *A. rabiei* populations from Australia. Node colors represent population membership proportional to the pie size. Node sizes are relatively scaled to logl.75n, where *n* is the number of samples in the nodes to reduce node overlap. Edge thickness (lines) represent minimum genetic distance between haplotype.

In total, 66 haplotypes were detected, of which 34 were detected just once (*n* = 1) (Supplementary Table 1). The detection frequency of each haplotype and the genetic relationships among them revealed by the seven SSR loci are presented in **Figure 3**. The most frequently detected haplotype, ARH01 accounted for 55.35 to 72.30% of the six regional population across all growing seasons (**Table 4**). In accordance with the overall gene diversity detected, the highest gene diversity was observed in Region 1 (*H_exp_* = 0.161), which contained the most unique and effective alleles (2.28 and 1.20, respectively). Gene diversity in Region 1 was significantly higher than in all other regions (*P* ≤ 0.001). The *eMLG* (9.00), *E.5* values (0.49), Ne (1.20), and Na (2.28) values were also highest for Region 1, indicating a more diverse population in this region compared to the other analyzed regions. However, the corrected Simpson’s genotypic diversity index (D) did not differ greatly among regions The mean low genotypic diversity (*D* = 0.57) indicated the consistent, low diversity detected within the entire Australian *A. rabiei* population (**Table 4**).

**Table 4:** The genetic structure of the population detected within each of the six growing regions.

Regions	N	MLG	*eMLG* ± SE	Na ± SE	Ne ± SE	*H_exp_* ± S.E	*E.5*	*D*	% of ARH01
1	18	9	9 ± 0.00	2.28 ± 0.47	1.20 ± 0.08	0.161 ± 0.05	0.498	0.555	55.55
2	233	38	6.8 ± 1.73	1.85 ± 0.45	1.05 ± 0.03	0.049 ± 0.03	0.269	0.555	63.09
3	130	21	5.38 ± 1.52	1.85 ± 0.40	1.04 ± 0.04	0.039 ± 0.03	0.312	0.592	72.30
4	92	18	6.37 ± 1.49	1.85 ± 0.34	1.07 ± 0.04	0.061 ± 0.03	0.382	0.592	60.86
5	56	16	7.78 ± 1.49	1.71 ± 0.28	1.06 ± 0.03	0.055 ± 0.02	0.393	0.554	55.35
6	69	16	6.82 ± 1.50	1.85 ± 0.45	1.03 ± 0.02	0.034 ± 0.01	0.362	0.652	62.31

*Mean *D* = 0.57. *N* = Number of individuals observed. MLG = Number of multilocus genotypes (MLG) observed. *eMLG* = The number of expected MLG at the smallest sample size based on rarefaction. SE = Standard error. Na = Number of different alleles (Kalinowski, 2005). Ne = Number of effective alleles (Kalinowski, 2005). *H*_*exp*_ = Nei’s unbiased gene diversity (Nei, 1978). *E.5* = Genotypic evenness (Pielou, 1975; Ludwig and Reynolds, 1988; Grünwald et al., 2003). *D* = Corrected Simpson’s Index (Simpson, 1949)*.

A total of 430 isolates were collected from the two widely adopted and “resistant” or “moderately resistant” host genotypes, Genesis090 and PBA HatTrick. Within these sub-populations, a total of 17 (*N* = 55) and 47 (*N* = 373) MLGs were observed on Genesis090 and PBA HatTrick, respectively (**Table 5**). Regardless of the host genotypes, the most frequently detected haplotype was ARH01, which accounted for 54 and 63% of the isolates detected on Genesis090 and PBA HatTrick, respectively (**Table 5**). Although more than five times the number of isolates were collected from PBA HatTrick than Genesis090, no significant difference in gene and genotypic diversity measures (*P* ≤ 0.45) was detected among the isolate groups. Furthermore, genetic diversity were all low (**Table 5**).

**Table 5:** The genetic structure of the *A. rabiei* population detected on two widely adopted host genotypes.

Hosts	*N*	MLG	eMLG ± SE	Na ± SE	Ne ± SE	*H_exp_* ± SE	*E.5*	D	% of ARH01
Genesis090	57	17	4.91 ± 1.32	1.85 ± 0.45	1.06 ± 0.03	0.052 ± 0.02	0.37	0.662	57.89
PBA HatTrick	373	47	4.28 ± 1.37	2.28 ± 0.42	1.05 ± 0.03	0.048 ± 0.02	0.26	0.575	64.61

*N = Number of individuals observed. MLG = Number of multilocus genotypes (MLG) observed. *eMLG* = The number of expected MLG at the smallest sample size based on rarefaction. SE = Standard error. Na = Number of different alleles (Kalinowski, 2005). Ne = Number of effective alleles (Kalinowski, 2005). *H_exp_* = Nei’s unbiased gene diversity (Nei, 1978). *E.5* = Genotypic evenness (Pielou, 1975; Ludwig and Reynolds, 1988; Grünwald et al., 2003). *D* = Corrected Simpson’s Index (Simpson, 1949)*.

#### No Evidence of Temporal or Spacial Population Shift

Although some differences in gene diversity (*H_exp_*) were observed between the 1st and 3rd years of sampling, increasing from 0.024 to 0.053 at Location A, and from 0.024 to 0.059 at Location B, these changes were not significantly different to those detected in the 2nd year of sampling. Also, the frequency of the ARH01 haplotype remained almost static at each independently sampled geographical location, A and B, and across the 3 years of sampling (ranging from 58.62 to 66.67%; **Figure 2**). Thus, no evidence of either a temporal or spacial population shift in SSR diversity was observed over the period of the study, at the epidemic locations sampled (**Table 6**).

**Table 6:** The genetic structure of the population detected within each of the six growing regions.

Year	Population locations (*N*)	MLG	eMLG ± SE	Na ± SE	Ne ± SE	*H_exp_* ± S.E	*E.5*	*D*	% of ARH01
2013	A (48)	12	8.86 ± 1.27	1.42 ± 0.29	1.02 ± 0.02	0.024 ± 0.018	0.382	0.544	66.67
	B (48)	12	8.86 ± 1.27	1.43 ± 0.29	1.02 ± 0.02	0.024 ± 0.018	0.382	0.540	66.66
2014	A (72)	21	10.51 ± 1.81	1.42 ± 0.29	1.03 ± 0.03	0.031 ± 0.021	0.308	0.584	63.88
	B (72)	21	10.51 ± 1.81	1.43 ± 0.29	1.03 ± 0.02	0.031 ± 0.021	0.308	0.630	63.88
2015	A (42)	10	8.15 ± 1.05	1.71 ± 0.47	1.06 ± 0.02	0.053 ± 0.029	0.418	0.630	66.66
	B (29)	9	8.867 ± 9.0	1.43 ± 0.20	1.06 ± 0.03	0.059 ± 0.028	0.491	0.576	58.62

*N = Number of individuals observed. MLG = Number of multilocus genotypes (MLG) observed. *eMLG* = The number of expected MLG at the smallest sample size based on rarefaction. SE = Standard error. Na = Number of different alleles (Kalinowski, 2005). Ne = Number of effective alleles (Kalinowski, 2005). *H_exp_* = Nei’s unbiased gene diversity (Nei, 1978). *E.5* = Genotypic evenness (Pielou, 1975; Ludwig and Reynolds, 1988; Grünwald et al., 2003). *D* = Corrected Simpson’s Index (Simpson, 1949)*.

#### Pathogenic Population Structure and Highest Risk Isolates

Among the 260 isolates assessed, 54 (21%) were highly aggressive and categorized into highly aggressive pathogenicity groups. Of these, 62% belonged to pathogenicity group 1, 2% belonged to pathogenicity group 2, 13% belonged to pathogenicity group 3 and 23% belonged to pathogenicity group 4 (Supplementary Table 2).

**FIGURE 4:**
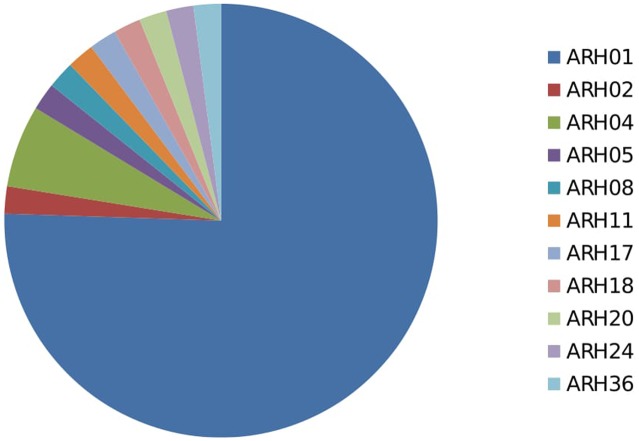
Proportion of highly aggressive isolates from different haplotype group.

Among the 54 highly aggressive isolates identified, 75% belonged to haplotype ARH01, far more than detected in any other haplotype group, and recovered across all of the growing regions and hosts assessed. Far lower frequencies of the highly aggressive isolates were detected within other haplotype groups (**Figure 4**; Supplementary Table 3).

## Discussion

Together with the strategic application of fungicides, chickpea production is reliant on the host containing the optimal combination of *A. rabiei* resistance alleles for timely recognition and defense initiation. Meanwhile, the pathogen population is under constant selective pressure to alter, to reproduce and spread new versions of itself that are able to survive in new environments, evade detection by the host and potentially overcome early host defenses (Pandey et al., 2016). In a clonal population, this occurs through opportunistic mutation and spread of the fittest and most widely adaptable isolates (Messer et al., 2016). Given the clonal nature of *A. rabiei* in Australia (Leo et al., 2015), it is unsurprising that we have detected a limited number of haplotypes that occur frequently throughout the chickpea growing regions, independent of host genotype.

### Population Structure: SSRs

The overall low genetic diversity found within the comprehensive assessment of the Australian *A. rabiei* population in this study is a common finding with Leo et al. (2015). Even if compatible mating types were present in the population, it seems likely that the expansion of this population to date has occurred through clonal means, perhaps due to forces suppressing recombination such as an initial imbalance in the mating type ratio of the founder isolates. The life cycles of many fungal species alternate between asexual and sexual multiplication (Hawker, 2016; Laloi et al., 2016) and asexual reproduction is often the major reproductive mechanism during epidemics to quickly increase the frequency of fit individuals (Laloi et al., 2016).

Within the Australian *A. rabiei* population, the large haplotype group (ARH01) occurs at extremely high frequencies of up to 63% of the entire population within a region. This haplotype likely established during an initial founder effect (Rivas et al., 2004) due to specific fitness characteristics that enabled survival at the point of introduction. The isolates then likely spread to other growing regions through infected seed distribution (Galloway and MacLeod, 2003). Subsequently, isolates that were highly adapted to survive in the range of agro-geographical regions and able to overcome host resistance proliferated through clonal propagation, causing severe disease epidemics when optimal climatic conditions prevailed. The resultant genetic and genotypic diversities observed in the Australian *A. rabiei* population reflect this founder effect, whereby the establishment and success of the pathogen has occurred through an available niche, provided by the abundance of susceptible host and an optimal environment. The number of founder events that have occurred for *A. rabiei* in Australia is unknown, but potentially the increasing frequencies of several haplotypes other than ARH01 in the population is an indication of slow evolution of other groups of highly adapted isolates, which should be monitored for increases in highly aggressive isolate frequency.

The genotypic diversity detected among isolates recovered from Genesis090 was not significantly different to that detected among those recovered from PBA HatTrick. Whilst it is likely that the host genotype would contribute to shaping the structure of the pathogen population (Jones and Dangl, 2006; Ley et al., 2006), the number of isolates assessed over the time period in the current study may not have been sufficient to visualize this phenomenon. Given the relatively short period for potential adaptation (<40 years), the clonal Australian population may still be experiencing the original founder effects. More in-depth investigation is required to determine if host factors are contributing to population adaptation. This might be through tracking of specific isolates over time and observations of the host defense responses that are instigated within each of the hosts under investigation. A similar, but smaller scale study was previously conducted by Leo et al. (2016), who found some host-specific differences in defense-related gene expressions. Expression of differential host defense responses to specific isolate populations might also identify factors that impact on survival and reproduction of specific populations and hence inform management strategies within growing regions where particular hosts are grown (Hollomon and Brent, 2009; Bertolini et al., 2012; Ali et al., 2016).

The non-significant differences detected in genotypic diversity over time at both of the locations assessed in New South Wales was unsurprising given the clonal nature of the pathogen and the short period since introduction to Australia. In a similar study, no significant changes were observed over 3 years within a *Mycosphaerella graminicola* population, DNA fingerprints were used to identify colones produced through asexual reproduction, suggesting and genetic stability of fungus was proposed (Chen et al., 1994).

Although overall seemingly stable and despite being clonal, we cannot ignore the potential for the existing population to change and evolve rapidly in response to an external factor (Messer et al., 2016). Rapid evolution of the wheat pathogen *Zymoseptoria tritici* was determined to be due to clustering of transposable elements leading to generation of extensive rearrangements and multiple independent gene losses (Hartmann et al., 2017). Rapid population changes may also occur through selective sweeps, potentially linked to host genotypes and/or chemical controls, resulting in “adaptive walk” and genetic shift among a limited number of frequently occurring haplotypes (Orr, 1998; Messer et al., 2016). Evidence of this may become more apparent as the industry adopts new resistant host genotypes such as PBA Seamer, which will need careful monitoring for pathogen population shift (Pulse Australia, 2017).

### The Pathogenic Population Structure

Evaluating *A. rabiei* populations on a set of differentials with different levels of resistance is useful for monitoring aggressiveness changes and to identify the most aggressive isolates. These isolates are required for selective breeding and, potentially, disease management strategies, particularly if differential factors underpinning aggressiveness are able to be dissected. Despite the SSR clonal composition, a similar wide diversity in aggressiveness was detected within the Australian population as previously detected elsewhere (Iqbal et al., 2004; Benzohra et al., 2011; Atik et al., 2013; Mahiout et al., 2015). Highly aggressive isolates were able to cause differential disease severities across a host set including two of the most widely adopted cultivars that underpin the Australian chickpea industry, Genesis090 and PBA HAtTrick. Genesis090 was introduced from ICARDA, Syria, where it was tested as FLIP94-090C, while PBA HatTrick is a cross of *cv.* Jimbour and the resistant Iranian landrace ICC14903 (Pulse Australia, 2017). However, these host genotypes have in recent years experienced an increase in susceptibility to *A. rabiei* (Moore et al., 2016), in keeping with our observation of increased frequencies of highly aggressive isolates within the population.

Increased aggressiveness within the Australian *A. rabiei* population was highlighted by the recent first observation of pycnidia formation on ICC3996, one of the resistance pillars of the chickpea breeding program, in field trials (Moore et al., 2016). Consequently, erosion of resistance in host genotypes that contain alleles from this source is highly likely as the adapted highly aggressive isolates spread and most likely become more frequent. Other sources of resistance will be necessary in the immediate future to underpin the chickpea breeding programs in Australia. Such material should be selected based on ability to resist the diversity of the pathogen population since different genetic mechanisms are likely to be controlling aggressiveness between the different pathogenicity groups (Hamid and Strange, 2000). Although the most destructive isolates detected in this study were of Pathogenicity Group 4, we must remember that this classification was based on disease severity on ICC3996. It is highly likely that differential reactions would occur on other resistance sources and that these should be more fully characterized for their own disease reactions to the representative pathogen population before being used within the breeding program.

## Conclusion

Within the adapted clonal groups detected in this study, we can surmise that isolates were selected by their ability to overcome resistance within the widely adopted “resistant” host genotypes such as Genesis090 and PBA HatTrick. This would help to explain the occurrence of a greater frequency of highly aggressive isolates within the ARH01 haplotype group, creating “super isolates” of the very highest pathogenicity ranking able to survive in many locations and on a wide range of host genotypes. These isolates represent the very highest risk to the Australian chickpea industry. However, several factors must be considered when selecting accessions that appear “resistant” to these: (1) This study suggests rapid changes in aggressiveness of the pathogen population, (2) only one mating type of *A. rabiei* has been detected in Australia but, if both mating types were present, the sexual reproductive cycle may quickly become active to create a recombinant pool population, and (3) isolate sampling and testing is limited by time and resources leading to the possibility of missing some important aggressive isolates. To extend stability of resistance, our growers must maintain their best practice in farming systems including growing clean seed, a minimum of 3-year rotations, effective distances between chickpea crops and fungicide spray regimes. Meanwhile, further studies are required to better understand the genetics of resistance in order to develop host genotypes with different resistance gene combinations, to potentially reduce selective adaptation of the pathogen.

## Author Contributions

YM assessed the molecular structure of the *A. rabiei* population and constructed the manuscript. PS assessed the pathogenic structure of the *A. rabiei* population and drafted the manuscript. JD, KH, KM, CL, SK, JB, and AL contributed to analyses and manuscript production. RF supervised the research and edited the manuscript.

## Conflict of Interest Statement

The authors declare that the research was conducted in the absence of any commercial or financial relationships that could be construed as a potential conflict of interest.

## Footnotes

1 http://faostat3.fao.org
